# miARma-Seq: a comprehensive tool for miRNA, mRNA and circRNA analysis

**DOI:** 10.1038/srep25749

**Published:** 2016-05-11

**Authors:** Eduardo Andrés-León, Rocío Núñez-Torres, Ana M. Rojas

**Affiliations:** 1 Instituto de Biomedicina de Sevilla (IBIS), Hospital Universitario Virgen del Rocío/CSIC/Universidad de Sevilla, Computational Biology and Bioinformatics Group, Seville, Spain

## Abstract

Large-scale RNAseq has substantially changed the transcriptomics field, as it enables an unprecedented amount of high resolution data to be acquired. However, the analysis of these data still poses a challenge to the research community. Many tools have been developed to overcome this problem, and to facilitate the study of miRNA expression profiles and those of their target genes. While a few of these enable both kinds of analysis to be performed, they also present certain limitations in terms of their requirements and/or the restrictions on data uploading. To avoid these restraints, we have developed a suite that offers the identification of miRNA, mRNA and circRNAs that can be applied to any sequenced organism. Additionally, it enables differential expression, miRNA-mRNA target prediction and/or functional analysis. The miARma-Seq pipeline is presented as a stand-alone tool that is both easy to install and flexible in terms of its use, and that brings together well-established software in a single bundle. Our suite can analyze a large number of samples due to its multithread design. By testing miARma-Seq in validated datasets, we demonstrate here the benefits that can be gained from this tool by making it readily accessible to the research community.

The development of high-throughput next-generation sequencing (NGS) technologies has revolutionized the transcriptomics field, paving the way for large-scale RNA sequencing (RNA-Seq)^1^. RNA-Seq can not only be used to study genome-wide transcription but also, it offers the ability to discover new genes and transcripts^2^ or to identify additional elements, such as new non-coding RNAs, small interfering RNAs (siRNAs), small nucleolar RNAs (snoRNAs) and micro-RNAs (miRNA). Recently, a new class of RNAs has been described, called circRNAs^3^, that are characterized by their ability to form circular RNA through a covalent linkage at the ends of a single RNA molecule. These circRNAs seem to participate in the regulation of gene expression, acting as regulators of miRNAs by specific binding to them. The appearance of these new regulatory molecules has led to the development of new tools for the identification of circRNAs, also through RNA-Seq experiments^4^.

There are two important aspects of RNA-Seq experiments, the vast amount of data generated in this kind of study, and the ability to extract and interpret biologically relevant information. These issues are particularly relevant since transcriptomics data analysis can easily become an important experimental bottleneck, especially given the additional constraints that both RNA-Seq and miRNA-Seq analyses impose. Indeed, the combination of different statistical and bioinformatics tools with many customizable parameters often makes such analysis difficult for non-experienced researchers. In addition, the use of different tools may involve time-consuming installations, usually requiring human intervention to proceed to the next step. To alleviate this problem, several tools have been generated for gene expression analysis, like ExpressionPlot^5^, GENE-counter^6^, RobiNA^7^, TCW^8^, Grape RNA-Seq^9^ or MAP-RSeq^10^. In addition, another set of tools focuses on the analysis of miRNA expression profiles, such as DSAP^11^, miRanalyzer^12^, miRExpress^13^, miRNAkey^14^, iMir^15^, CAP-miRSeq^16^, mirTools 2.0^17^ or sRNAtoolbox^18^. Moreover, a few tools have been implemented to perform both RNA-Seq and miRNA-Seq analysis, such as wapRNA^19^, eRNA^20^, BioVLAB-MMIA-NGS^21^ or Omics Pipe^22^. Other available methods integrating several software enabling different type of NGS analyses are GALAXY (https://galaxyproject.org/), QuasR^23^, RAP^24^, Subread/edgeR^25^, while others provide a collection of modules to process files, like the ViennaNGS^26^ suite.

Although extremely valuable, the main disadvantage of these tools is that, with some exceptions, they often still rely on manual installation procedures and further human input, steps that have proven difficult to automate. There are also other issues that hamper their wider diffusion and implementation: i) some of the tools have been designed to work on web-based platforms with the consequent restriction on data upload or limited offer of parameter’s choice (i.e Galaxy, RAP^24^, BioVLAB-MMIA-NGS^21^, or DSAP^11^); ii) the analysis pipelines implemented have rigid workflows, so users cannot start the analyses at different steps of the pipeline (i.e. RAP^24^, BioVLAB-MMIA-NGS^21^); iii) some of these tools have a large list of pre-requisites for local installation that complicates their use by less experienced researchers (i.e.: Cap-miRSEq^16^, Omics Pipe^22^, iMir^15^, Galaxy, ExpressionPlot^5^); iv) the analysis is usually restricted to a few selected model organisms (i.e. QuasR^23^, ExpressionPlot^5^, BioVLAB-MMIA-NGS^21^), and iiv) some tools uses in-house code which has not been extensively tested in the NGS community (i.e. Grape RNA-Seq^9^ or ExpressionPlot^5^). In addition, to our knowledge, none of these tools has implemented a pipeline for the analysis of circRNAs.

With these limitations in mind, we have developed a comprehensive pipeline analysis suite called “miARma-Seq”, which stands for *miRNA-Seq And RNA-Seq Multiprocess Analysis*, that is designed to identify mRNAs, miRNAs and circRNAs, as well as for differential expression, target prediction and functional analysis. Most importantly, it can be applied to any sequenced organism, and it can be initiated at any step of the workflow.

## Results

### miARma-Seq main features

**Figure 1:**
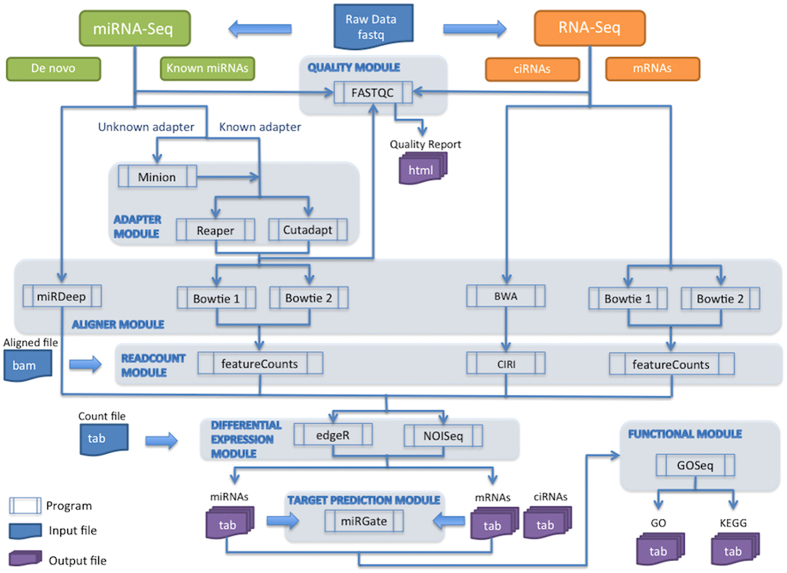
miARma-Seq pipeline workflow. An overview of the modular design of the pipeline. Main modules are indicated by gray background. Output files are indicated by purple background.

The most important aspect of the suite is that it is a stand-alone tool that is both easy to install and extremely flexible in terms of its use as compared to other methods (Supplementary Table S1). It brings together well-established software in a single bundle, allowing a complete analysis from raw data (Fig. 1). All the capabilities can be easily and simultaneously enabled at will, regardless the step in the workflow. We have tested miARma-Seq using different published datasets of miRNAs, mRNAs, and circRNAs to illustrate some of the tool capabilities and to show that the tool works as expected (Supplementary Tables S2 and S3, Supplementary Results).

### miARma-Seq computational performance

We computed the performance in terms of time used to run each analysis (Table 1). To illustrate miARma-Seq’s capabilities in the analysis of miRNA-Seq data, we processed the SRR873382 sample from the GSE47602 experiment with our pipeline in a standard computer, with 8 GB of RAM memory and a 1.7 GHz CPU. The sample selected has over 35 million reads and it was analyzed using the multithread option (4 threads). Total analysis of the sample took 19 minutes and it included: a quality analysis (2 min); pre-processing of the sample that included adapter removal with Reaper^27^ (6 min); alignment against a reference genome with Bowtie 1^28^ (8 min); and summarizing of the read counts (3 min). Similarly, the SRR873382 sample was also processed with miARma-Seq to identify novel miRNAs (DeNovo analysis). The total analysis of the sample took 48 minutes including: a quality analysis (2 min), pre-processing of the sample (<1 min); alignment against reference genomes; and summarizing the read counts with miRDeep 2^29^ (45 min).

The performance of miARma-Seq for RNA-Seq data analysis was also evaluated using a paired-end SRR1039508 sample of nearly 44 million reads from the GSE52778 experiment and using 4 threads on a standard computer as describe above. The total analysis of the sample took 172 minutes and it included: a quality analysis (6 min), alignment against reference genomes with TopHat^30^/Bowtie 2^31^ (161 min) and the summarizing of the read counts (5 min).

In order to demonstrate how miARma-Seq can be implemented to analyze circRNAs we processed the SRR1051292 sample from the GSE49321 experiment on a standard computer set up as indicated above. The sample selected was a paired-end sample with over 7 million reads and it was analyzed using the multithread option (4 threads). The total analysis of the sample took 48 minutes and it included: a quality analysis (3 min), an alignment against a reference genome with BWA^32^ (35 min) and the summarizing of the read counts with CIRI^4^ (10 min).

### Evaluation of miARma-Seq

Proper validation studies to produce meaningful statistics cannot be conducted with the available experimental data, which in its current state does not provide complete information of false positives (FP) or false negatives (FN). Therefore, precision cannot be calculated. Nonetheless, we calculated correlations with previous studies as a proxy to control whether or not the tool was performing as expected.

In this regard, we used miARma-Seq to analyze three case scenarios. In miRNA transcriptome analyses, we analyzed the expression analysis of regulated miRNAs from a time course experiment under different hypoxic conditions, and miRNA novel detection as well. We next run mRNA genome-wide analyses, and finally we performed analyses to detect circRNAs from RNA-seq data. All these analyses showed a strong overlapping with previous studies (Supplementary Tables S2 and S3, Supplementary Fig. SF1), confirming that the pipeline is working as it should be expected.

## Discussion

High throughput NGS technology is now being widely employed for many purposes in scientific research. Although the vast majority of the existing tools are very valuable and efficient in analyzing such data (Supplementary Table S1), most of them are difficult to be tested by users without basic programming and computing skills. In this regard, the NGS computational-based community has made a tremendous effort to make these tools usable. For instance, software’s interoperability is no longer an issue, as most of them enable local installation for different operative systems (Supplementary Table S1).

A widely used strategy to optimize usability is the development of web-based versions of particular tools, which in one hand comes with a cost in terms of several restrictions. For instance, most of web-based systems (i.e., Galaxy, RAP^24^), may present issues intrinsically related to web-traffic (i.e. bandwith limits, data privacy policies affecting users, and/or queue saturation, among others). Even when these systems enable their local installation, the process can be very demanding in terms of required expertise to handle the installation (i.e., Galaxy). They also can be limited in the number of analyses or data uploads (i.e., RAP^24^), or they may be built upon in-house code, which has not been extensively tested in the NGS community (i.e., DSAP^11^).

However, the main issue that hampers the difusion of these methods, relies on difficulties related to overcome software dependencies when installing, which usually requires advanced computing skills. All together, these restrictions make the full potential of any tool too difficult to be properly exploited by non-experienced users (QuasR^23^, Subread/edgeR^25^, Cap-MirSeq^16^, Omics pipe^22^, etc).

Here, we present miARma-Seq, a comprehensive pipeline analysis for RNA-Seq and miRNA-Seq data suitable to identify mRNAs, miRNAs and circRNAs in any organism with a sequenced genome, and to analyze their Differential Expression. This tool aims to resolve some of the main problems that researchers might encounter when analyzing high throughput sequencing data: i) integrated capabilities that allow data from both miRNA and mRNA expression experiments to be analyzed; ii) easy installation, without having to check otherwise tedious requirements that make the analysis more difficult for non-experienced researchers; iii) no restrictions regarding the reference organism as it is not restricted to the analysis of only a few model organisms but rather, it can be used with any reference organism as long as its genome has been sequenced; iv) flexibility in terms of fitting any experimental design, allowing the user to start the analysis at different steps of the pipeline; v) Speed of execution, allowing the pipeline to be executed in a standard computer or in a cluster environment (profiting from its parallelizing properties); vi) reliability, as the tool performs the analysis with the most of the standard tools available; vii) Wide coverage, which not only includes miRNA-Seq and RNA-Seq data analysis to identify miRNAs, mRNAs or circRNAs but also, the possibility to carry out differential expression analysis, target prediction and functional analysis, and to predict novel miRNAs. We have tested miARma-Seq using different published datasets and the results obtained correlated well with the original sources.

A particularly interesting characteristic of miARma-Seq, is the possibility to detect and identify circRNAs from RNA-Seq data, and to analyze their differential expression. The recent description of these molecules means only a few tools have been developed to detect circRNAs and to our knowledge, no pipeline has included this kind of analysis.

One of the main goals of our pipeline is to offer a fast and a flexible tool for miRNA and RNA-Seq data analysis. In this sense, we demonstrated that using a standard computer that can be found in any research laboratory, the median time required to detect known miRNAs, novel miRNAs, mRNAs and circRNAs in a sample is 19, 48, 172 and 48 minutes, respectively. Obviously, these are illustrative processing times, since the time required will depend on aspects such as the depth of the sample, the tool selected for analysis, the steps included in the analysis and the computer employed.

In contrast to some web-based tools, miARma-Seq is designed for local execution on small computers or at big computing infrastructures that take advantage of multithread analysis, which is especially recommended to rapidly analyze huge amounts of data.

This kind of analysis is usually faster than that offered by web-based tools, since one of the main problems in these tools is that the processing time depends on the number of jobs pending in the queue and on the server’s capacity.

In order to make it easier to use our pipeline, we have developed a stand-alone tool with extremely easy to install software (downloading only one unique file), with minimum software requirements. Therefore, we consider that miARma-Seq offers the speed of a stand-alone tool with the ease of a web-based tool.

In conclusion, miARma-Seq is a powerful and very flexible tool for transcriptomics (miRNA, mRNA and circRNAs), which offers additional capabilities including the identification of differentially expressed entities, miRNA-mRNA target prediction, or functional analysis (Supplementary Figs S1 and S2). The miARma-Seq pipeline can perform a fast and reliable analysis of a large number of samples due to its multithread design and the established quality of the software included (Supplementary Table S2). The tool can be used by a wide range of users, from those having little experience in programming and computing in both installation and usage to advanced users more accustomed to the command line handling. It runs in three different operative systems, and it has been tested for reliance on software updates.

Notably, it successfully provided well-correlated data when compared with validated and published results obtained from different transcriptomics analyses (Supplementary Tables S2 and S3), demonstrating the utility of this tool for the research community.

## Methods

### Code availability

A stand-alone version has been developed to minimize software requirements and to simplify its installation on any computer, while allowing non-expert users to perform complex analyses. The tool has been tested in the latest Fedora 23, Centos 7.2, Ubuntu (from 10.4 to 14.04) and Debian jessie. It has been also tested in Apple computers from 10.9 to 10.11.

The tool’s performance is not affected by updates of the included software and the current version contains the most updated software. Nonetheless, previous versions of miARma-Seq are also available with outdated versions of the included software, so the user can compare results (i.e. to check reproducibility among different versions of the software). In the case of CIRI^4^, we offer the choice of methods via configuration file.

The stable code in miARma-Seq is freely available at https://sourceforge.net/projects/miarma/.

The development code in miARma-Seq is freely available at https://bitbucket.org/cbbio/miarma/src.

A complete guide for the installation, analysis of miRNA, mRNA and circRNA data is provided, along with different examples of its use, at http://miarmaseq.cbbio.es/.

### Description and implementation

To facilitate the installation process, we provide three different possibilities of installing the software for Mac and Linux (Source code, four Docker images, and a virtual Image), and two possibilities for Windows 7, 8, 8.1 and 10 (four Docker images and a virtual Image). Detailed information on how to install is available here (http://miarmaseq.cbbio.es/installation).

The miARma-Seq tool was implemented as a combination of Perl and R scripts for Unix environments. To further simplify its use, the miARma-Seq pipeline has preconfigured and customized parameters that can be executed with a simple command line program and a configuration file. Nevertheless, miARma-Seq can be used by more experienced bioinformaticians, since Perl modules are freely available and can be configured in the custom pipelines.

The miARma-Seq tool has a highly flexible modular structure designed to perform the different stages of analysis (Fig. 1). This structure offers the possibility to start the analysis at any point in the workflow by simply providing a configuration file. This characteristic is most useful for the analysis of pre-processed data from public databases, which usually implies aligned bam or tab separated read-count files. In addition, the configuration file allows the user to define which software to use at each step. Alternative software can be used as a single election or simultaneously. Nevertheless, the resources included in miARma-Seq have been selected by comparing and evaluating software widely used in RNA-Seq and miRNA-Seq data analysis^33^,^34^,^35^,^36^, or based on the advantages offered for the analysis (i.e.: adapter prediction or replicate simulation).

Although the analysis can start at any point in the pipeline, the typical input files in sequenced transcriptome experiments are fastq files, a common format used in miRNA, mRNA and circRNAs studies. These files are processed by miARma-Seq with specific software for each data type, while the main analysis stages are shared.

### Quality assessment and pre-processing

Initialy, all the fastq files are submitted for quality evaluation using FastQC software (http://www.bioinformatics.babraham.ac.uk/projects/fastqc/), which provides a detailed report of the quality of the reads. In order to customize the pre-processing of the reads, miARma-Seq offers different tools for customization (for details see Supplementary methods). Cutadapt^37^ and Reaper^27^ are included for trimming purposes. The Minion software^27^ enables to perform an adapter sequence prediction. In addition, miARma-Seq also includes an in-house tool to remove a specific number of nucleotides from the 3′ or 5′ end that usually contain low quality information.

As a result of the read trimming, miARma-Seq generates plots with the length distribution of the reads in each sample (Supplementary Fig. S2). After pre-processing, miARma-Seq offers the possibility of performing additional quality control analyses with FastQC to assess the quality of the processed reads.

### Alignment

This stage differs according to the kind of the data to be analyzed. For miRNA expression studies, read alignment can be performed with Bowtie^28^, Bowtie2^31^ or both. In addition, miARma-Seq includes mirDeep2^29^ to predict novel mirnas (for details see Supplementary methods). TopHat^30^ is available, which can also execute the miRNA alignment software. In both cases, miARma-Seq offers the possibility to construct genome indices from fasta files offering the possibility to analyze any organism with a sequenced genome. For circRNAs detection the BWA aligner^32^ is available (for details see Supplementary methods).

### Entity quantification

FeatureCounts^38^ was implemented in miARma-Seq to summarize reads. In addition, novel miRNAs can also be quantified using mirDeep2^29^. The quantification of circRNA reads is achieved with CIRI^4^ (for details see Supplementary methods). A tab separated read-count file is generated as an output at this stage.

### Exploratory analysis of the data

This analysis generates an exhaustive PDF report that allows every aspect of the processed data to be inspected in detail. This report includes a boxplot and a density plot of the normalized and non-normalized data, which facilitates the inspection of the read distribution, as well as a multidimensional scaling (MDS) plot, a principal component analysis (PCA) plot, a heatmap (Supplementary Fig. S2), and clustering plots.

### Differential expression analysis

Two alternative software tools that can be combined: edgeR^39^ and Noiseq^40^ (for details see Supplementary methods). As a result of the analysis, easy to explore tabulated files are generated with the DE elements (Supplementary Fig. S2) and the main statistic values, as well as exploratory plots for each experimental condition evaluated, such as volcano or expression plots (Supplementary Fig. S2).

### Target prediction

Through miRGate, miARma-Seq offers a miRNA-mRNA target prediction module for DE miRNAs or genes^41^. miRGate is a database containing novel predicted miRNA-mRNA pairs that are calculated using well-established algorithms, including miRanda^42^, Pita^43^, RNAhybrid^44^ or MicroTar^45^, among others. In addition, miRGate includes experimental validated miRNA-mRNA pairs that provide miARma-Seq a highly reliable tool for gene target prediction. Notably, miARma-Seq not only provides the potential targets of the DE miRNAs or mRNAs detected but if both sets of data are provided, negative correlations between miRNAs and mRNAs can be performed to extract DE mRNAs targeted by DE miRNAs. Thus, a detailed report is generated by miARma-Seq that includes the DE miRNAs and their corresponding mRNA targets, as well as statistical information on the number of tools in which there is an agreement for each prediction and the experimental validated targets.

### Gene list analysis

A gene list analysis is implemented in miARma-Seq using the Goseq tool^46^. Goseq allows gene ontologies (GO) and metabolic pathways (KEGG) to be identified in genome-wide expression analyses. This enables the user to understand the biological processes affected in the experiment, either a metabolic pathway or a cellular complex, and it reduces complexity by highlighting the biological processes. This analysis generates a tabulated file (excel compatible) with information regarding the GO terms identified and their corresponding categories, ordered for relevance (p-value) of the up- and down-regulated entities.

### Final summary report

Transcriptome expression analysis involves long, and multistep process that generates a wide variety of important information. To help understand all this relevant data, miARma-Seq generates an easy to read summary of the entire analysis requested. This summary is an excel compatible file that includes a section for each step of the analysis, presenting the main statistics: number of reads processed, total identified entities, number of aligned reads, number of DE entities, etc. An example of this summary report is presented in the Supplementary Fig. S2.

### Datasets used to compute performance of miARma-Seq

#### miRNA transcriptome analysis

In order to evaluate the ability of miARma-Seq to detect, identify and assess the Differentitally expressed (DE) miRNAs in time-course experiments, a miRNA expression dataset^47^ was analysed (GEO experiment GSE47602). Briefly, this experiment measures miRNAs regulated under different hypoxic conditions in the MCF7 cell line. It contains 2 replicates in normal conditions and 2 replicates taken after 16, 32 and 48 hours in hypoxic conditions. In addition to known miRNAs, miARma-Seq allows novel miRNAs to be identified and analyzed. The miRNA-Seq data from the hypoxic cell indicated above was used to detect novel miRNAs.

#### mRNA genome-wide expression analysis

The expression data available from the GSE52778 GEO experiment was used to test the capacity of our pipeline for RNA-Seq analysis^48^. This experiment contains mRNA samples obtained from four primary human airway smooth muscle cell lines untreated or treated with dexamethasone, albuterol, dexamethasone+albuterol. To facilitate the understanding of the pipeline, only samples treated with dexamethasone and control samples (3 replicates for each condition) were analyzed.

#### Detection of circRNAs from RNA-Seq data

The detection and identification of circRNAs from RNA-Seq data is a recent incorporation into the RNA-Seq analysis setting. To verify our circRNA analysis implementation, we used the data available from the GEO GSE49321 experiment^33^. Briefly, different cell types were used to identify the circRNAs expressed specifically in seven samples from HEK293T cells.

## Additional Information

**How to cite this article**: Andrés-León, E. *et al*. miARma-Seq: a comprehensive tool for miRNA, mRNA and circRNA analysis. *Sci. Rep.* **6**, 25749; doi: 10.1038/srep25749 (2016).

## Supplementary Material

Supplementary Information

## Figures and Tables

**Table 1:** Performance of miARma-Seq tool.

Type	EXP	Sample	Scope	Reads	T*	Q*	PRE*	ALIGN*	SUMM*
miRNA	GSE47602	SRR873382	D	34686701	19	2	6 (Reaper)	8 (Bowtie 1)	3 (FeatureCounts)
miRNA	GSE47603	SRR873383	I, dN	34686701	48	2	<1 (mirDeep2)	45 (mirDeep2 uses Bowtie 1 for the alignment)	
mRNA	GSE52778	SRR1039508	mD	45871042	172	6	**	161 (TopHat/Bowtie 2)	5 (FeatureCounts)
circRNAs	GSE49321	SRR1051292	cD	6767745	48	3	**	35 (BWA)	10 (CIRI)

The analyses have been performed in an average computer with 8 GB of RAM memory, 1.7 GHz CPU, and 4 threads. **EXP**: Experiment identifier. **SCOPE**: D stands for “detection” of known miRNAS, ID indicates “identification”, dN indicates “de novo” prediction of miRNAS, mD indicates “detection” of mRNAS, cD indicates “detection” of circRNAS. **Reads:** Number of reads per sample. **T**: Total analyses time, **Q**: Quality analyses time (FastQC). **PRE**: Preprocessing time (Software used). **ALIGN**: Alignment time (Software used). **SUMM**: Summarization of read counts time (Software used).

^*^Time in minutes.

^**^The use of pre-processing stage will depend on the sequencing process.
