# Corrigendum: miARma-Seq: a comprehensive tool for miRNA, mRNA and circRNA analysis

**DOI:** 10.1038/srep46928

**Published:** 2018-01-08

**Authors:** Eduardo Andrés-León, Rocío Núñez-Torres, Ana M. Rojas

Scientific Reports 6: Article number: 2574910.1038/srep25749; published online: 05 11 2016; updated: 01 08 2018

This Article contains out-of-date links to the miARma-Seq workflow webpages in the Methods section. Therefore, in the ‘Code availability’ subsection:

“A complete guide for the installation, analysis of miRNA, mRNA and circRNA data is provided, along with different examples of its use, at http://miarmaseq.cbbio.es/”.

should read:

“A complete guide for the installation, analysis of miRNA, mRNA and circRNA data is provided, along with different examples of its use, at http://miarmaseq.idoproteins.com/”.

In the ‘Description and implementation’ subsection:

“Detailed information on how to install is available here ( http://miarmaseq.cbbio.es/installation)”.

should read:

“Detailed information on how to install is available here ( http://miarmaseq.idoproteins.com/installation)”.

