# Supplementary material for: miARma-Seq: a comprehensive tool for miRNA, mRNA and circRNA analysis
Source: Sci Rep. 2016 May 11;6:25749. doi: 10.1038/srep25749 (PMC4863143; doi:10.1038/srep25749)
Supplement: Supplementary Information [file srep25749-s1.pdf]

# SUPPLEMENTARY MATERIAL

## miARma-Seq: a comprehensive tool for miRNA, mRNA and circRNA analysis

**Eduardo Andrés-León, Rocío Núñez-Torres, and Ana M Rojas.**

# Table of Content

**SUPPLEMENTARY FIGURES**.......... 2
**Supplementary Figure 1.** .......... 2
**Supplementary Figure 2.** .......... 4
**SUPPLEMENTARY TABLES**.......... 5
**Supplementary Table 1.** Most used methods in transcriptomics.......... 5
**Supplementary Table 2. Identification and DE of miRNAs and mRNAs**.......... 8
**Supplementary Table 3.** Identification of circRNAs.......... 8
**SUPPLEMENTARY TEXT**.......... 9
**METHODS**.......... 9
Quality assessment and pre-processing.......... 9
Alignment .......... 10
Entity quantification .......... 10
Differential expression analysis.......... 10
**RESULTS AND DISCUSSION**.......... 12
miRNA transcriptome analysis.......... 12
Genome-wide expression analysis of mRNA.......... 13
Detection of circRNAs from RNA-Seq data.......... 13
**SUPPLEMENTARY REFERENCES** .......... 14

# SUPPLEMENTARY FIGURES

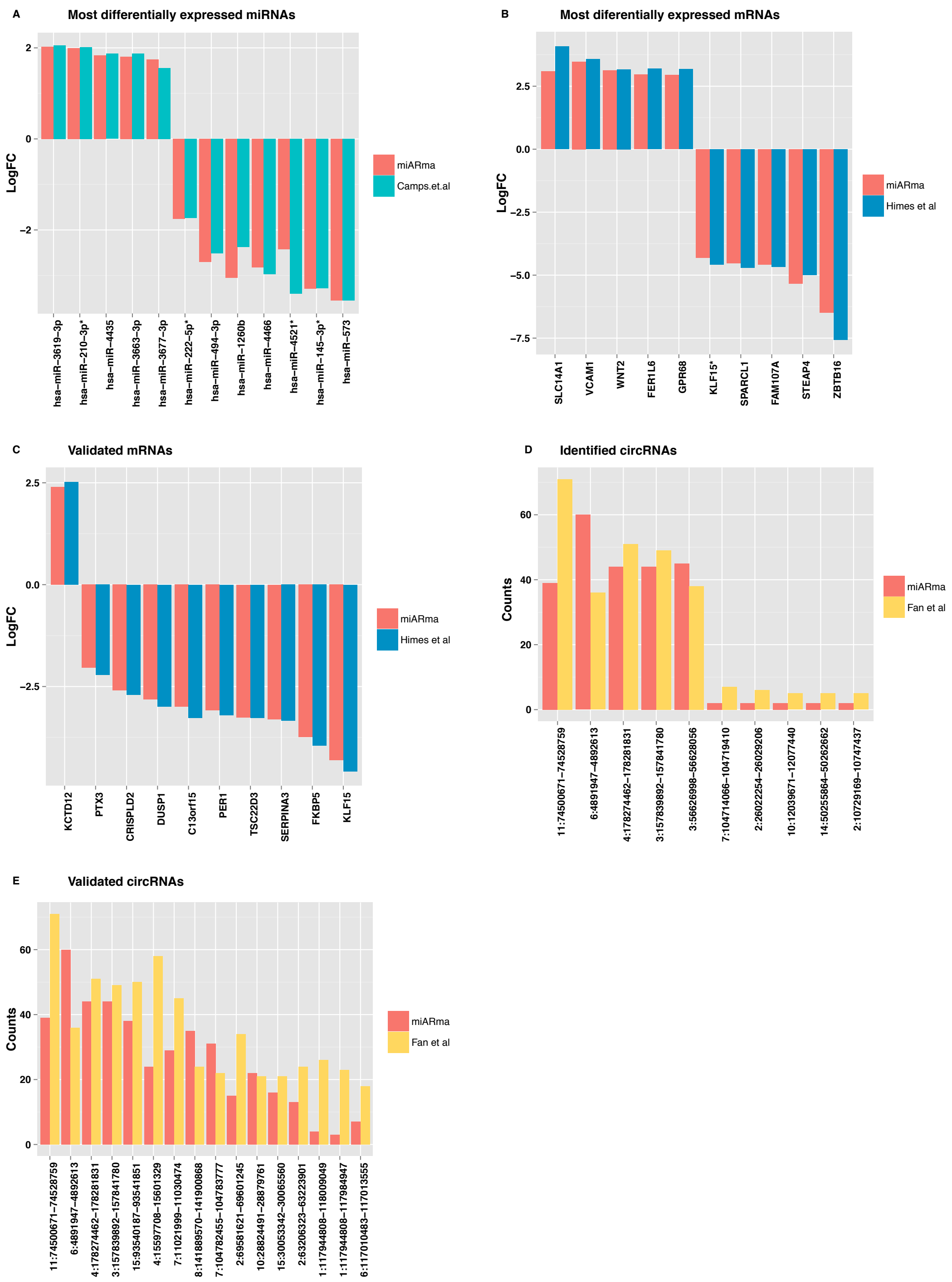


**Supplementary Figure 1.**

**Figure 1. Comparison between the results obtained using the miARma-Seq pipeline and those reported previously.** (A) Logarithmic fold change (logFC) of ten

miRNAs showing extreme differences in Fold change (FC) determined by miARma-Seq and reported elsewhere[1]. The experimentally validated miRNAs are indicated with an asterisk. (B) logFC values of ten mRNAs showing extreme differences in FC determined by miARma-Seq and reported elsewhere[2]. (C) logFC values of the experimentally validated mRNAs determined by miARma-Seq and reported elsewhere[2]. (D) Ten circRNAs showing extreme differences in the number of mapped reads that were identified by miARma-Seq and those reported elsewhere[3]. (E) Sixteen experimentally validated circRNAs determined by miARma-Seq and reported elsewhere [3].

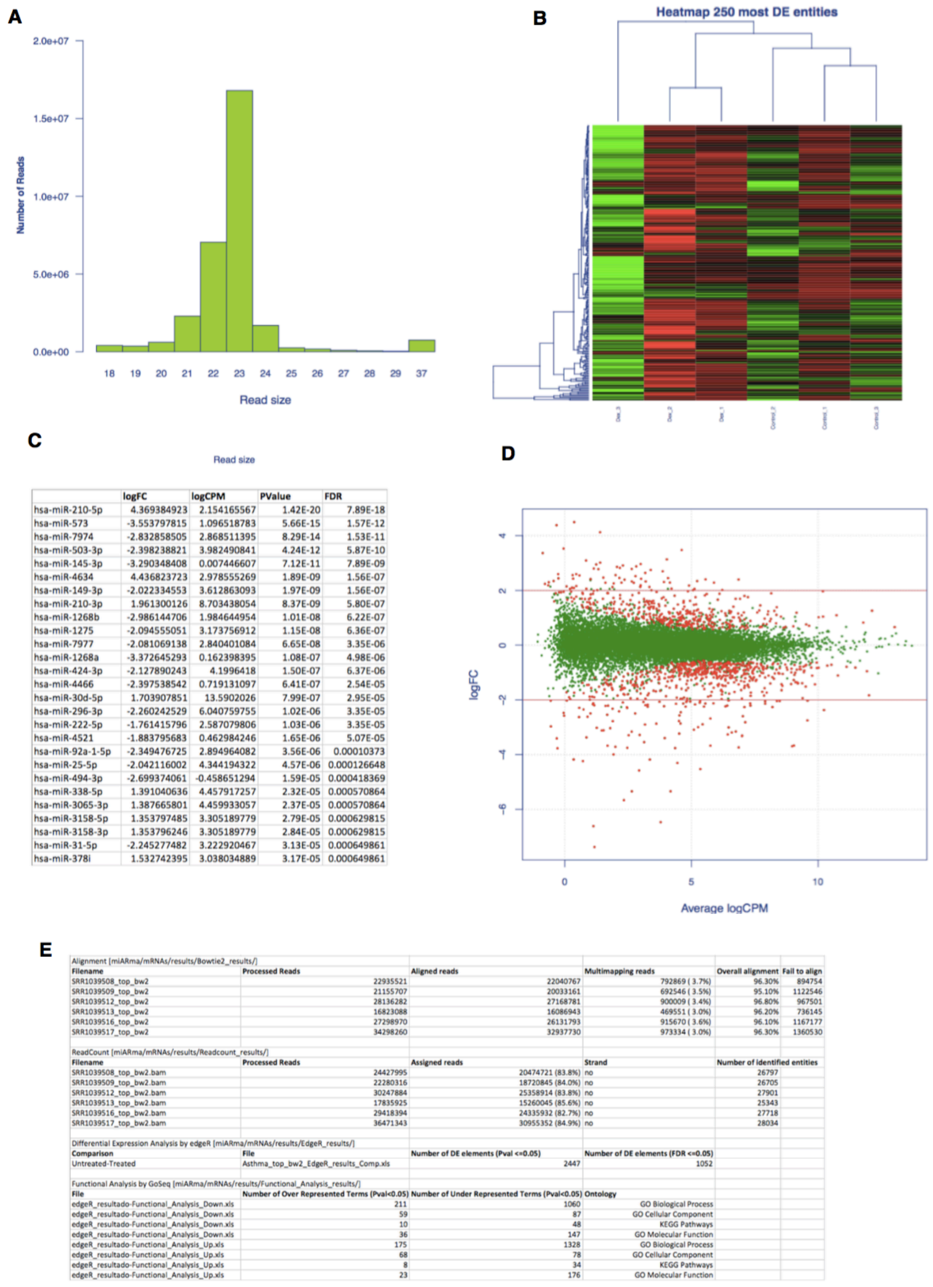


| | logFC | logCPM | PValue | FDR |
|---|---|---|---|---|
| hsa-miR-210-5p | 4.369384923 | 2.154165567 | 1.42E-20 | 7.89E-18 |
| hsa-miR-573 | -3.553797815 | 1.096518783 | 5.66E-15 | 1.57E-12 |
| hsa-miR-7974 | -2.832858505 | 2.868511395 | 8.29E-14 | 1.53E-11 |
| hsa-miR-503-3p | -2.398238821 | 3.982490841 | 4.24E-12 | 5.87E-10 |
| hsa-miR-145-3p | -3.290348408 | 0.007446607 | 7.12E-11 | 7.89E-09 |
| hsa-miR-4634 | 4.436823723 | 2.978555269 | 1.89E-09 | 1.56E-07 |
| hsa-miR-149-3p | -2.022334553 | 3.612863093 | 1.97E-09 | 1.56E-07 |
| hsa-miR-210-3p | 1.961300126 | 8.703438054 | 8.37E-09 | 5.80E-07 |
| hsa-miR-1268b | -2.986144706 | 1.984644954 | 1.01E-08 | 6.22E-07 |
| hsa-miR-1275 | -2.094555051 | 3.173756912 | 1.15E-08 | 6.36E-07 |
| hsa-miR-7977 | -2.081069138 | 2.840401084 | 6.65E-08 | 3.35E-06 |
| hsa-miR-1268a | -3.372645293 | 0.162398395 | 1.08E-07 | 4.98E-06 |
| hsa-miR-424-3p | -2.127890243 | 4.1996418 | 1.50E-07 | 6.37E-06 |
| hsa-miR-4466 | -2.397538542 | 0.719131097 | 6.41E-07 | 2.54E-05 |
| hsa-miR-30d-5p | 1.703907851 | 13.5902026 | 7.99E-07 | 2.95E-05 |
| hsa-miR-296-3p | -2.260242529 | 6.040759755 | 1.02E-06 | 3.35E-05 |
| hsa-miR-222-5p | -1.761415796 | 2.587079806 | 1.03E-06 | 3.35E-05 |
| hsa-miR-4521 | -1.883795683 | 0.462984246 | 1.65E-06 | 5.07E-05 |
| hsa-miR-92a-1-5p | -2.349476725 | 2.894964082 | 3.56E-06 | 0.00010373 |
| hsa-miR-25-5p | -2.042116002 | 4.344194322 | 4.57E-06 | 0.000126648 |
| hsa-miR-494-3p | -2.699374061 | -0.458651294 | 1.59E-05 | 0.000418369 |
| hsa-miR-338-5p | 1.391040636 | 4.457917257 | 2.32E-05 | 0.000570864 |
| hsa-miR-3065-3p | 1.387665801 | 4.459933057 | 2.37E-05 | 0.000570864 |
| hsa-miR-3158-5p | 1.353797485 | 3.305189779 | 2.79E-05 | 0.000629815 |
| hsa-miR-3158-3p | 1.353796246 | 3.305189779 | 2.84E-05 | 0.000629815 |
| hsa-miR-31-5p | -2.245277482 | 3.222920467 | 3.13E-05 | 0.000649861 |
| hsa-miR-378i | 1.532742395 | 3.038034889 | 3.17E-05 | 0.000649861 |

| | | | | | |
|---|---|---|---|---|---|
| Alignment [miARma/mRNAs/results/Bowtie2_results/] | | | | | |
| **Filename** | **Processed Reads** | **Aligned reads** | **Multimapping reads** | **Overall alignment** | **Fail to align** |
| SRR1039508_top_bw2 | 22935521 | 22040767 | 792869 ( 3.7%) | 96.30% | 894754 |
| SRR1039509_top_bw2 | 21155707 | 20033161 | 692546 ( 3.5%) | 95.10% | 1122546 |
| SRR1039512_top_bw2 | 28136282 | 27168781 | 900009 ( 3.4%) | 96.80% | 967501 |
| SRR1039513_top_bw2 | 16823088 | 16086943 | 469551 ( 3.0%) | 96.20% | 736145 |
| SRR1039516_top_bw2 | 27298970 | 26131793 | 915670 ( 3.6%) | 96.10% | 1167177 |
| SRR1039517_top_bw2 | 34298260 | 32937730 | 973334 ( 3.0%) | 96.30% | 1360530 |
| | | | | | |
| ReadCount [miARma/mRNAs/results/Readcount_results/] | | | | | |
| **Filename** | **Processed Reads** | **Assigned reads** | **Strand** | **Number of identified entities** | |
| SRR1039508_top_bw2.bam | 24427995 | 20474721 (83.8%) | no | 26797 | |
| SRR1039509_top_bw2.bam | 22280316 | 18720845 (84.0%) | no | 26705 | |
| SRR1039512_top_bw2.bam | 30247884 | 25358914 (83.8%) | no | 27901 | |
| SRR1039513_top_bw2.bam | 17835925 | 15260045 (85.6%) | no | 25343 | |
| SRR1039516_top_bw2.bam | 29418394 | 24335932 (82.7%) | no | 27718 | |
| SRR1039517_top_bw2.bam | 36471343 | 30955352 (84.9%) | no | 28034 | |
| | | | | | |
| Differential Expression Analysis by edgeR [miARma/mRNAs/results/EdgeR_results/] | | | | | |
| **Comparison** | **File** | **Number of DE elements (Pval <=0.05)** | **Number of DE elements (FDR <=0.05)** | | |
| Untreated-Treated | Asthma_top_bw2_EdgeR_results_Comp.xls | 2447 | 1052 | | |
| | | | | | |
| Functional Analysis by GoSeq [miARma/mRNAs/results/Functional_Analysis_results/] | | | | | |
| **File** | **Number of Over Represented Terms (Pval<0.05)** | **Number of Under Represented Terms (Pval<0.05)** | **Ontology** | | |
| edgeR_resultado-Functional_Analysis_Down.xls | 211 | 1060 | GO Biological Process | | |
| edgeR_resultado-Functional_Analysis_Down.xls | 59 | 87 | GO Cellular Component | | |
| edgeR_resultado-Functional_Analysis_Down.xls | 10 | 48 | KEGG Pathways | | |
| edgeR_resultado-Functional_Analysis_Down.xls | 36 | 147 | GO Molecular Function | | |
| edgeR_resultado-Functional_Analysis_Up.xls | 175 | 1328 | GO Biological Process | | |
| edgeR_resultado-Functional_Analysis_Up.xls | 68 | 78 | GO Cellular Component | | |
| edgeR_resultado-Functional_Analysis_Up.xls | 8 | 34 | KEGG Pathways | | |
| edgeR_resultado-Functional_Analysis_Up.xls | 23 | 176 | GO Molecular Function | | |

**Supplementary Figure 2.**

**Examples of miARma-Seq output files.** (A) A histogram of the read length after pre-processing of the sample. (B) Heatmap of the most strongly expressed elements according to Fold Change (the 250 differentially expressed elements). (C) Differential expression analysis output table. (D) Expression plot. (E) Summary report with the main statistics of the analysis.

# SUPPLEMENTARY TABLES

**Supplementary Table 1.** Most used methods in transcriptomics.

| Method | Data type | Software included | OS | Requisites | Potential issues | Expert Know. Use | Expert Know. Install | USAGE type | Type of analyses | Model organism |
|---|---|---|---|---|---|---|---|---|---|---|
| miARmaSeq | miRNA<br>mRNA<br>circRNA | **Q:** Fastqc<br>**T:**CutAdapt,Minion,Reaper;<br>**A:**Bowtie1,Bowtie2,TopHat, BWA;<br>**EQ:** FeatureCounts, Ciri,mirDeep2;<br>**Others:** RNAfold,Samtools;<br>**DE**: EdgeR,NoiSeq;<br>**Fx**: Goseq;<br>**Target:** miRGate (3 types of analyses) | 3 | Java, R and Perl. | N/A | NONE | NONE | Conf. file | **Differential Expression**: mirnas, mRNAs, circRNAs;<br>**Identification**: circrnas; miRNAs,<br>**Enrichements** mirnas-mrnas target prediction (3 methods); **miRNA de novo pred** | Any |
| QuasR (Gaidatzis et al, 2015) | miRNA<br>mRNA | **Q**: QcReport,;<br>**A:** Bowtie;<br>**EQ**: Qcount | 3 | R language | For R programmers. Except bowtie is (1) | Adv. R language | YES | R script | **Quality, alignment**, R processing to further use R programs for **Differential expression, SNPs, ChipSe**q, etc. | 21 organisms stored in Bioconductor |
| Subread/edgeR (Liao et al, 2013) | miRNA<br>mRNA | **Aligner**: subread;<br>**EQ**: featureCounts. | 3 | R language | For programmers. | Adv. R language & command line | YES | R script<br>Bash CLI | **Differential Expression**: mirnas, mRNAs,<br>**Enrichements, SNPs** | Any |

| **Method** | **Data type** | **Software included** | **OS** | **Requisites** | **Potential issues** | **Expert Know. Use** | **Expert Know. Install** | **USAGE type** | **Type of analyses** | **Model organism** |
|---|---|---|---|---|---|---|---|---|---|---|
| Cap-miRSEq (Sun et al 2014) | miRNA | **Q**: Fastqc;<br>T: Cutadapt;<br>**A**: Bbowtie,miRdeep;<br>**SNPs**: GATK;<br>**Q**:Htseq;<br>**DE:** EdgeR | 3 | Many in local installation | (2), (3), (4). | Adv.comp. skills | YES | Conf. file | **Differential Expression**: mirnas; **de novo prediction**, SNPs in miRNAs | N/A |
| Omics Pipe (Fisch et al, 2015) | miRNA mRNA | AWS of Amazon | 3 | Many in local installation | (2), (3), (4). | NONE | YES | Conf. file | **RNASeq, Whole Exomes, Whole genomes, Chipseq** | N/A |
| ViennaNGS (Wolfinger et al, 2015) | Aligned data | Modules to process files | 1 | Bedtools, Bedgraptht obigwig, R, Samtools | (7) | Adv.Perl language | YES | Perl scripts | N/A | N/A |
| Expression plot (Friedman, et al 2011) | mRNA | **Q**: in-house code;<br>**A:** bowtie<br>**DE**: Deseq | 1 | Many | (1), (2) | NONE | YES | web | **RNASeq and microarrays** | Human, mouse, and rat |
| Gene-Counter (Cumbie et al, 2011) | mRNA | **A:** Cashx, bowtie, bwa;<br>**DE**: NBPSeq, EdgeR, Deseq;<br>**Fx**: GoRich | 1 | Perl, MySQL, R, as well as C++ software (CASHX) | (2) | NONE | YES | Conf. file | **RNAseq** | Any |
| Imir (Giurato et al, 2013) | miRNAs | **A**: bowtie;<br>**DE**: deseq; denovo | 1 | Java, R , Python, and Perl. | (2) | Adv. Comp. skills | YES | Python scripts Perl scripts | **Differential Expression: mirnas, miRNA-mRNA target prediction** | N/A |

Supplementary Table S1 cont.

| **Method** | **Data type** | **Software included** | **OS** | **Requisites** | **Potential issues** | **Expert Know. Use** | **Expert Know. Install** | **USAGE type** | **Type of analyses** | **Model organism** |
|---|---|---|---|---|---|---|---|---|---|---|
| BioVLAB-MMIA-NGS (Chae et al, 2015) | miRNA mRNA Arrays | **A:** mirdeep1,tophat-cufflinks; **DE:** edgeR, limma, **FX**:David; **miRNa-mRNA target**:MMIA | 2 | JRE for the browser | (2), (5) | NONE | N/A | web | **Differential Expression**: mirnas, mRNAs; **Enrichements**; **mirnas-mrnas target prediction; miRNA de novo pred** | Human,mouse,macaca, and rice |
| GALAXY (https://galaxyproject.org/) | miRNA mRNA | Sveral tools (hundreds) | 2 | several | (2),(5) | NO | YES | web | all | Any |
| RAP (D'Antonio et al, 2015) | mRNA | **Q:** Fastqc,NGS QC toolkit; **A**:Tophat; **Alternative Splicing**: SpliceTrap; **Chimeric:** Chimerscan; **DE**: Cuffdiff2, DESeq | 2 | An Account | (3), (5), (6) | NO | N/A | web | **Differential Expression**: mRNAs; **Chimera**; **Poliadenylation**; **Splicing** | Eukaryotes |
| Dsap (Huang et al 2010) | miRNAs | in-house code software | 2 | N/A | Only works for illumina | Basic skills | N/A | web | **miRNAs** | N/A |

**Features of different methods**. This is an illustrative table and other alternative methods are not included. Only representative features are indicated. **Software included**: **Q:** quality, **T:** trimming, **A:** Aligner, **EQ**: EntityQuatification, **DE:** Differential expression, **Fx:** Functional analyses, **Target**: mRNA-mirNA; **OS: Operative System**, 3 (Mac, Lin & Win), 1 is Unix, 2 is Web-based. **Potential issues: (1)** methods not widely tested in the NGS scientific community, (2) Several dependencies, (3) Data privacy issues, (4) Potential payment requested, (5) Bandwidth issues and/or queue saturation, (6) restrictions on number of files and/or data size, (7) this tool is a collection of core modules to process files; **Expert Know. Use**, Expert knowledge required to use it: Adv.: Advanced, Comp.: computational; **Expert Know. Install:** Expert knowledge required for installation. N/A stands for not available/not identified.

## Supplementary Table 2. Identification and DE of miRNAs and mRNAs

| Experiment | Time course or total | # of RNAs | FDR | # of RNAs identified as DE in original work | % overlap between procedures | Pearson coefficient of correlation | p-value of Pearson Coeffcient of Correlation | % overlap with validated experimentally |
|---|---|---|---|---|---|---|---|---|
| miRNA transcriptome[5] | total at 16h (1) | 12 | 0.05 | 5 | 100 | 0.97 | 0.0076 | 100 |
| | total at 32h (1) | 94 | 0.05 | 86 | 83.7 | 0.98 | <2.2 e -16 | 100 |
| | total at 48h (1) | 135 | 0.05 | 119 | 85.7 | 0.98 | <2.2 e -16 | 100 |
| | **total** (1 ) | **154*** | **0.05** | 137* | **85.4** | **N/C** | **N/C** | **100** |
| | **Total** ( 2) | **554** | **N/A** | **501** | **91.8** | **-** | **-** | **100** |
| mRNA transcriptome[14] | **Total (1)** | **1052** | **<0. 05** | **316** | **98.4** | **0.99** | **<2.2 e -16** | **100** |

Identification and **DE** of miRNAs and mRNAs. **PLEASE NOTE that these values are illustrative, as precision cannot be computed**. **The recall must be taken with caution.** Those are comparisons with original works as internal control of the tool. (1) indicates Differentially Expressed (DE) RNAs. *indicates unique entities as some miRNAs can be DE in various time courses; (2) Total number of expressed miRNAs. N/C (Not calculated): correlations cannot be calculated as certain miRNAs can be detected

## **Supplementary Table 3.** Identification of circRNAs

| | According to # of reads* | # of RNAs identified | # of RNAs identified in original work | % overlapping | Pearson coefficient of correlation | p-value of Pearson Coefficient of Correlation | % overlap with experimentally validated circRNAs |
|---|---|---|---|---|---|---|---|
| | >1 | 229 | 141 | 61.9 | 0.81 | <2.2e -16 | 84.2 |
| CircRNAS[18] | >4* | 124 | 78 | 87.5 | 0.78 | 3.9 e-13 | |

Identification of circRNAs . * Recommended elsewhere[19] (>4). Out of these 19 validated circRNAs we have identified 16.

# SUPPLEMENTARY TEXT

## METHODS

### Quality assessment and pre-processing

Quality assesment is a standard and important procedure in NGS analysis, as it detects sequencing errors. On the other hand, low quality reads, low complexity bases (those biased in their nt composition), high number of N content, and high number of duplicated reads, are very important issues to consider before performing aligment and entity quantification steps. In this regard, miARma-Seq uses fastQC (http://www.bioinformatics.babraham.ac.uk/projects/fastqc/), a widely used method to perform quality checking.

Since miRNAs have an estimated size of 22 nucleotides, pre-processing of the samples is mandatory to remove adapter sequences and low-quality bases, which could affect the correct alignment of the reads and in particular, that of short sequences <50 bp. Cutadapt[4] and Reaper[5] are designed to trim the adapter sequence or part of it, as well as to perform additional filtering (low complexity).

One common problem when a user is dealing with data from public databases is the lack of experimental information, including the nature of the adapter sequences, which is essential information required by most trimming software. To avoid this problem, miARma-Seq implements the Minion software[5], which performs an adapter sequence prediction based on the most frequent sequences at the 3' end of the read. In this sense, miARma-Seq can automatically predict the adapter sequence even if this information is not provided, checking the data obtained with Minion. Besides, miARma- Seq uses Blat software (https://genome.ucsc.edu/cgi-bin/hgBlat) in order to rule out over-expressed biological sequences that can be identified as potential adapters. In this way, Cutadapt[4] or Reaper[5] can be provided with adapter sequences for the subsequent analysis.

In addition, miARma-Seq also includes an in-house tool to remove a specific number of nucleotides from the 3' or 5' end that usually contain low quality information meant to be used in miRNA, mRNA and circRNAs samples.

## Alignment

miARma-seq implements different aligners according to the kind of the data to be analyzed. For miRNAs, Bowtie1[6], Bowtie2[7] or both simultaneously can be used. According to Bowtie developers, for reads smaller than 50bp (usually whay is found in miRNASeq experiments), Bowtie1 is recommended.

For novel miRNA prediction, miARma-Seq implements mirDeep2[8], which collects non-aligned miRNA-Seq data to predict novel miRNAs based on different features, such as the ability of a pre-miRNA to form a hairpin structure or to follow the pattern of Dicer processing.

In the case of RNASeq, TopHat[9] is provided as the main tool for mRNA alignment and exon junction calculation. TopHat in miARma-Seq is implemented to use Bowtie1[6], Bowtie2[7] or both. As explained before, for reads shorter than 50bp, bowtie1[6] is recommended. In any other case, Bowtie2[7] is highly recommended.

For circRNA processing, the BWA aligner[10] was implemented in miARma-Seq (as recommend[11]) since BWA-MEM implements a local alignment that maximizes the exact matches. In any case, a standard binary alignment file (BAM) is generated.

## Entity quantification

Because the number of reads from a RNA transcript (counts) is proportional to the abundance of that transcript[12], the number of counts in biological entities must be summarized. For that purpose featureCount (ref) is implemented. It is a highly efficient general-purpose read summarization program that counts mapped reads for genomic features. For novel miRNA quantification, miRDeep2 (ref) provides this feature and in the case of circRNAs, miARma-Seq implements the CIRI[11] tool, which is based on paired chiastic clipping signal detection in BAM files, quantifies the reads, and also performs a systematic filtering to remove false positives.

## Differential expression analysis

Most expression studies focus on the identification of entities differentially expressed (DE) between two different experimental conditions. Of these two valuable tools (edgeR[13] and Noiseq[14]) to identify DE elements, edgeR is a widely employed tool that

not only identifies DE elements between two experimental conditions but it also performs more complicated comparisons. This is a key issue in studies analyzing time series or combined drug effects. Therefore, unlike most of the available analytic tools that can only compare two experimental conditions, miARma-Seq can identify DE elements in any kind of experiment design. Another common difficulty in DE analysis is the lack of biological or technical replicates to perform the analysis. Although it is strongly recommended to avoid this situation due to the poor reliability of the results, experimental issues occasionally force researchers to perform their experiments without replicates due to the lack of samples or the poor quality of the replicates. In these situations, miARma-Seq implements Noiseq, which allows technical replicates to be simulated in order to increase the reliability of the results. Weakly expressed elements may also distort the results as elements with low read counts might not reveal true biological information. Filtering these weakly expressed elements has been proposed as an appropriate way to avoid this problem. Thus, miARma-Seq offers the user the possibility of filtering the data to introduce a minimum cut-off value among other parameters. Normalization is also a key step to facilitate sample comparison in this kind of analysis and by default, miARma-Seq normalizes the data, although users can skip this step or even chose the normalization method that best fits their data.

## RESULTS AND DISCUSSION

To check whether the tool works as it should, we compared our results with previous studies. Please note, that in all cases **recall should be taken with caution**, as it is not possible to calculate precision because the information of False positives (FP) and False Negatives (FN) is incomplete in the experimental datasets. In experimental studies is rare to find the 100% of results experimentally validated. Usually only 10-20 selected targets within the potential positive results are validated.

### miRNA transcriptome analysis

In order to evaluate the ability of miARma-Seq **to detect, identify and assess the DE** of miRNAs, a miRNA expression dataset[1] was analysed (GEO experiment GSE47602). Briefly, this experiment measures miRNAs regulated under different hypoxic conditions in the MCF7 cell line. It contains 2 replicates in normal conditions and 2 replicates taken after 16, 32 and 48 hours in hypoxic conditions. A total of 554 miRNAs were identified, with a 91.8% overlap with previous results. The Differential expression (DE) analysis of the three experimental conditions compared to control samples identified many DE miRNAs (FDR<0.05) at different time points. The results correlated very well with the previously reported data: Pearson correlation at 16 h, 0.97 (P.val =0.0076); 0.99 at 32 h (P.val < 2.2e-16); and 0.98 at 48 (P.val < 2.2e-16: Supplementary Fig. S1). In addition, miARma-Seq correctly identified 100% of the DE miRNAs experimentally validated by qPCR. The differences in the results are mainly due to the different miRBase annotation database employed and the weakly expressed elements, which were filtered out in our analysis. Indeed, we find a strong correlation (>0.97) for the expression of the common miRNAs in each experimental condition.

The miRNA-Seq data from the hypoxic cell indicated above was used to detect novel miRNAs. A total of 113 novel miRNAs were identified. Of which, 9, 11 and 14 were Differentially Expressed after 16, 32 and 48 hours of hypoxia, respectively (Supplementary Fig. S1).

In addition, we inspected the expression of experimentally validated miRNAs and we verified that every validated miRNA was detected in our pipeline, ascribing a similar value to those reported with *in vitro* techniques. These results demonstrate the suitability of our pipeline to detect and analyze known miRNAs.

Furthermore, the miARma-Seq pipeline includes the miRDeep2 tool that predicts novel miRNAs and applying this DeNovo analysis to a published dataset identified more than one hundred novel miRNAs. Unfortunately, no information regarding validated novel miRNAs was available to compare with our results.

## Genome-wide expression analysis of mRNA

The miARma-Seq pipeline was also applied to **genome-wide expression** data to **identify mRNAs** in a set of primary human airway smooth muscle cell lines treated with dexamethasone[2], (Supplementary Table S2, Supplementary Fig. S1).

The miARma-Seq platform identified a total of 1052 DE genes in the samples (FDR<0.05), detecting 98.48% of those found in the original study and with a correlation of 0.99 (P.val < 2.2e-16). The numbers of identified DE genes are in agreement with those obtained in well-established software for similar comparative analyses (for details see[15,16]).

The vast majority of the DE mRNAs described were detected previously. In addition, the correlation between the expression of common mRNAs in both experiments was almost perfect (Supplementary Table S2), even though another tool was that used originally to summarize the reads and for the DE analysis, Cufflinks[17]. This strong consistency between the results obtained with both tools, Cuffdiff and EdgeR[13], is not surprising given that they are both robust[15,16]. Furthermore, all DE mRNAs experimentally validated were detected with our pipeline, supporting the validity of the results obtained with miARma-Seq.

## Detection of circRNAs from RNA-Seq data

The detection and identification of circRNAs from RNA-Seq data is a recent incorporation into the RNA-Seq analysis setting. To verify our circRNA analysis implementation, we used the data available from the GEO GSE49321 experiment [3].

The analysis of RNA-Seq data derived from seven samples of HEK293T cells[3] identified 229 circRNAs (Supplementary Table S3), detecting 61.9% of the circRNAs described in the original work. The highest and lowest numbers of reads identified in each circRNAs were compared (Supplementary Fig. S1), and we evaluated the experimentally validated circRNAs detected. A total of 16 out of 19 experimentally

validated circRNAs were identified by miARma-Seq (Supplementary Fig. S1, Supplementary Table S3).

Nevertheless, the overall correlation of the counts of common circRNAs was good (0.81, P.val <2.2e -16) as the tool identified most of the validated circRNAs (84.2%), supporting its use in our pipeline for circRNA detection.

The main differences in the circRNAs detected were derived from the filtering process during the analysis with CIRI. Notably, when we compared our results with the circRNAs previously reported to be expressed above a recommend threshold[18], our pipeline identified a total of 87.5% of the circRNAs. In addition, the dataset selected for validation comes from a single cell RNA-Seq experiment, which may be distinct to traditional RNA-Seq experiments, explaining the differences found between both sets of results. Nevertheless, the overall correlation of the expression values (counts) of common circRNAs was good as the tool identified most of the validated circRNAs, supporting its use in our pipeline for circRNA detection

# SUPPLEMENTARY REFERENCES


1 Camps, C. *et al.* Integrated analysis of microRNA and mRNA expression and association with HIF binding reveals the complexity of microRNA expression regulation under hypoxia. *Molecular cancer* **13**, 28, doi:10.1186/1476-4598-13-28 (2014).

2 Himes, B. E. *et al.* RNA-Seq transcriptome profiling identifies CRISPLD2 as a glucocorticoid responsive gene that modulates cytokine function in airway smooth muscle cells. *PloS one* **9**, e99625, doi:10.1371/journal.pone.0099625 (2014).

3 Fan, X. *et al.* Single-cell RNA-seq transcriptome analysis of linear and circular RNAs in mouse preimplantation embryos. *Genome biology* **16**, 148, doi:10.1186/s13059-015-0706-1 (2015).

4 Creighton, C. J., Nagaraja, A. K., Hanash, S. M., Matzuk, M. M. & Gunaratne, P. H. A bioinformatics tool for linking gene expression profiling results with public databases of microRNA target predictions. *Rna* **14**, 2290-2296, doi:10.1261/rna.1188208 (2008).

5 Davis, M. P., van Dongen, S., Abreu-Goodger, C., Bartonicek, N. & Enright, A. J. Kraken: a set of tools for quality control and analysis of high-throughput sequence data. *Methods* **63**, 41-49, doi:10.1016/j.ymeth.2013.06.027 (2013).

6 Langmead, B., Trapnell, C., Pop, M. & Salzberg, S. L. Ultrafast and memory-efficient alignment of short DNA sequences to the human genome. *Genome biology* **10**, R25, doi:10.1186/gb-2009-10-3-r25 (2009).
7 Langmead, B. & Salzberg, S. L. Fast gapped-read alignment with Bowtie 2. *Nature methods* **9**, 357-359, doi:10.1038/nmeth.1923 (2012).
8 Friedlander, M. R., Mackowiak, S. D., Li, N., Chen, W. & Rajewsky, N. miRDeep2 accurately identifies known and hundreds of novel microRNA genes in seven animal clades. *Nucleic acids research* **40**, 37-52, doi:10.1093/nar/gkr688 (2012).
9 Kim, D. *et al.* TopHat2: accurate alignment of transcriptomes in the presence of insertions, deletions and gene fusions. *Genome biology* **14**, R36, doi:10.1186/gb-2013-14-4-r36 (2013).
10 Li, H. & Durbin, R. Fast and accurate long-read alignment with Burrows-Wheeler transform. *Bioinformatics* **26**, 589-595, doi:10.1093/bioinformatics/btp698 (2010).
11 Gao, Y., Wang, J. & Zhao, F. CIRI: an efficient and unbiased algorithm for de novo circular RNA identification. *Genome biology* **16**, 4, doi:10.1186/s13059-014-0571-3 (2015).
12 Liao, Y., Smyth, G. K. & Shi, W. featureCounts: an efficient general purpose program for assigning sequence reads to genomic features. *Bioinformatics* **30**, 923-930, doi:10.1093/bioinformatics/btt656 (2014).
13 Robinson, M. D., McCarthy, D. J. & Smyth, G. K. edgeR: a Bioconductor package for differential expression analysis of digital gene expression data. *Bioinformatics* **26**, 139-140, doi:10.1093/bioinformatics/btp616 (2010).
14 Tarazona, S., Garcia-Alcalde, F., Dopazo, J., Ferrer, A. & Conesa, A. Differential expression in RNA-seq: a matter of depth. *Genome research* **21**, 2213-2223, doi:10.1101/gr.124321.111 (2011).
15 Nookaew, I. *et al.* A comprehensive comparison of RNA-Seq-based transcriptome analysis from reads to differential gene expression and cross-comparison with microarrays: a case study in Saccharomyces cerevisiae. *Nucleic acids research* **40**, 10084-10097, doi:10.1093/nar/gks804 (2012).
16 Zhang, Z. H. *et al.* A comparative study of techniques for differential expression analysis on RNA-Seq data. *PloS one* **9**, e103207, doi:10.1371/journal.pone.0103207 (2014).
17 Trapnell, C. *et al.* Transcript assembly and quantification by RNA-Seq reveals unannotated transcripts and isoform switching during cell differentiation. *Nat Biotechnol* **28**, 511-515, doi:10.1038/nbt.1621 (2010).
18 Anders, S. *et al.* Count-based differential expression analysis of RNA sequencing data using R and Bioconductor. *Nature protocols* **8**, 1765-1786, doi:10.1038/nprot.2013.099 (2013).
